# An fNIRS-based investigation of cerebral hemodynamic responses during verbal fluency task and n-back task in individuals with mild cognitive impairment

**DOI:** 10.3389/fneur.2025.1571964

**Published:** 2025-05-08

**Authors:** Can Duan, Yufei Chong, Jingyi Gong, Qingqing Wu, Jialing Sun, Chanjuan Zheng, Zhengliang Li, Lirong Xia, Zhen Cheng, Peiwen Zhang, Wenguang Xia

**Affiliations:** ^1^College of Chinese Medicine, Hubei University of Chinese Medicine, Wuhan, China; ^2^Department of Rehabilitation, Xinhua Hospital of Hubei University of Chinese Medicine (Hubei Provincial Hospital of Integrated Traditional Chinese and Western Medicine), Wuhan, China; ^3^Rehabilitation Department, The Affiliated Hospital of Hubei Provincial Government (Hubei Rehabilitation Hospital), Wuhan, China; ^4^Hubei Engineering Research Center of Neuromodulation Technology, Wuhan, China; ^5^Hubei Provincial Clinical Research Center for Stroke Rehabilitation of Integrated Traditional Chinese and Western Medicine, Wuhan, China

**Keywords:** functional near-infrared spectroscopy, mild cognitive impairment, verbal fluency task, working memory, neural degeneration

## Abstract

**Background:**

Early detection of mild cognitive impairment (MCI) is crucial for preventing Alzheimer’s disease (AD). This study aims to explore alterations in brain co-functional connectivity between cognitively healthy individuals and those with cognitive impairment during a verbal fluency task (VFT) using functional near-infrared spectroscopy (fNIRS). The investigation examines changes in brain activation patterns in both MCI patients and healthy controls during the VFT and 1-back task, and identifies correlations between cognitive function and brain activation areas using fNIRS technology.

**Methods:**

This study evaluated markers for screening MCI by performing the VFT and 1-back task using a 67-channel fNIRS to measure changes in oxyhemoglobin (HbO) levels in the bilateral prefrontal and temporal lobes of 108 healthy controls (HC) and 103 participants with MCI. The severity of patients’ symptoms was assessed using the Montreal Cognitive Assessment (MoCA) scale, neuropsychiatric symptoms were evaluated with the Symptom Checklist-90 (SCL-90), and sleep quality was assessed using the Pittsburgh Sleep Quality Index (PSQI).

**Results:**

Compared with the HC group, the MCI group showed a significant reduction in MoCA scores, with no significant differences in education level, PSQI, and SCL-90 scores. There was no significant difference in brain activation levels between the MCI and HC groups during the VFT. However, during the 1-back task, the MCI group exhibited significantly reduced activation levels in channels 33, 54, 49, and 47, as well as in the dorsolateral prefrontal cortex (DLPFC) and frontal eye fields (FEF). Moreover, the mean HbO levels in these channels, DLPFC, and FEF during the 1-back task were found to be significantly correlated with MoCA scores.

**Discussion:**

When performing the VFT and 1-back task, our study found that patients with MCI exhibited reduced brain activity levels in the DLPFC and FEF only during the 1-back task. This diminished task-induced brain activity was significantly positively correlated with MoCA scores and was less influenced by mental health and sleep quality. The 1-back task may be a more optimal paradigm for the early detection of MCI compared to the VFT.

## 1 Introduction

With the acceleration of population aging, the prevalence of mild cognitive impairment (MCI) and dementia has been on the rise, establishing itself as a substantial global public health concern. According to World Health Organization statistics, over 9.9 million new dementia cases are reported worldwide annually, resulting in an economic burden estimated at approximately 8.4 trillion yuan. By 2030, projections indicate that the total annual societal cost of dementia in China will surpass 3 trillion yuan (1), imposing a considerable strain on societal resources. Recent investigations have revealed that the prevalence of MCI among individuals aged 60 and above in China reaches 15.5% (2), with annual progression rates to dementia ranging between 10 and 15%, which is 3 to 10 times greater than that observed in cognitively normal elderly populations (3, 4). Most dementia patients identified in clinical contexts are found in the middle to advanced stages of the condition. Notably, MCI is regarded as a reversible condition and has been globally acknowledged as the most favorable phase for dementia prevention (2, 5). Consequently, the demand for more precise tools and diagnostic standards capable of facilitating the early detection of MCI has become increasingly critical. Identifying MCI at an earlier stage and fortifying preventive measures against dementia remain pivotal challenges and priorities within brain science research.

At present, the early diagnosis of MCI is primarily dependent on medical history, scales, body fluid analysis, and neuroimaging (6). Nevertheless, scale-based assessments are inherently subjective and may exhibit reduced accuracy, especially when administered repeatedly. Research on body fluid examinations has demonstrated that biomarkers such as amyloid *β*-42/40, total tau, phosphorylated tau-181 (p-tau181), and phosphorylated tau-217 (p-tau217) in cerebrospinal fluid exhibit certain sensitivity in the identification of MCI. However, the widespread application of these tests is constrained by their high cost, procedural complexity, and associated risks (7, 8). Neuroimaging, on the other hand, offers notable advantages for diagnosing MCI, particularly in terms of temporal and spatial resolution as well as anatomical precision. In recent years, common neuroimaging modalities have included functional magnetic resonance imaging (fMRI), positron emission tomography (9), magnetoencephalography (MEG), functional near-infrared spectroscopy (fNIRS), and electroencephalography (EEG). Among these techniques, fMRI remains the most extensively utilized diagnostic tool. However, its feasibility for early screening is hindered by high costs, time-intensive procedures, and the unavailability of necessary equipment in many grassroots institutions. fNIRS, a more recent non-invasive brain imaging technology, provides real-time insights into brain oxygenation and metabolism by leveraging the absorption and scattering properties of near-infrared light (600–900 nm) in biological tissues. This approach enables the functional state of the brain to be reflected and is particularly advantageous for populations unable to undergo fMRI (10). Compared to fMRI, fNIRS delivers superior temporal resolution, allowing for real-time monitoring and recording of cerebral blood flow and hemoglobin variations. Additionally, it objectively visualizes the spatiotemporal characteristics of neural activity through the neurovascular coupling mechanism (11–13).

**Figure 1:**
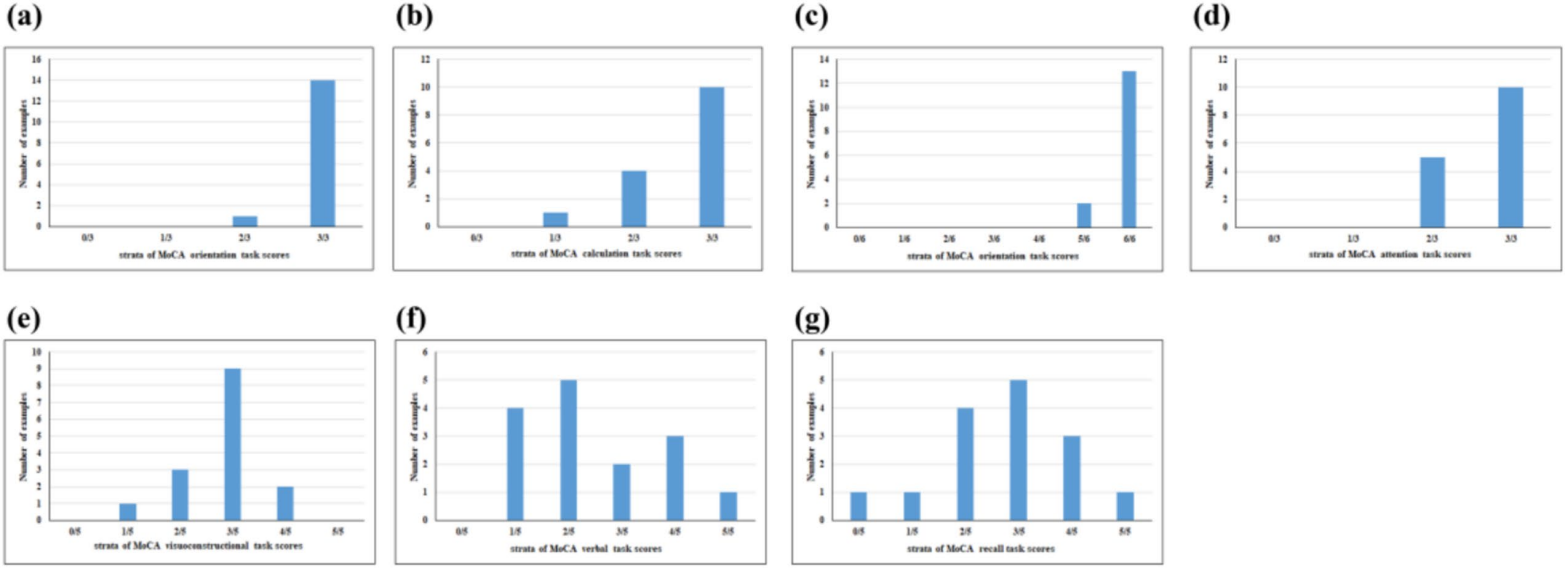
Proportion of participants with cognitive domain dysfunction of MoCA-assessed cognitive subscore cognitive domains included **(a)** naming, **(b)** calculation, **(c)** orientation, **(d)** attention, **(e)** visuoconstructional, **(f)** verbal, and **(g)** recall.

It has been identified by some scholars that verbal fluency functions as an effective predictor of cognitive decline (14). When compared to other cognitive domains, such as memory, verbal fluency has been deemed more reliable for the early detection of cognitive decline (15). A number of studies have demonstrated that verbal fluency task (VFT) are sensitive measures of cognitive dysfunction in MCI (16, 17). The VFT typically include two types of tasks: phonological fluency and semantic fluency (18). The phonological fluency task involves executive function, working memory, and phonological processing skills, which are closely related to left prefrontal function, usually by speaking words that begin with a specific letter or contain a specific Chinese character within a specified period of time (19, 20). The semantic fluency task is to speech words that belong to a certain semantic category, such as fruits, vegetables, animals, etc., within a specified amount of time (21). This process is related to semantic memory, vocabulary retrieval, and classification ability, which requires proper functioning of Wernicke’s region in the temporal lobe and left parietal lobe (22, 23). Both verbal fluency and semantic fluency have demonstrated efficacy in distinguishing healthy individuals from those with MCI and Alzheimer’s disease (AD) (24). Performance in verbal fluency tasks has been consistently observed to be poorer among MCI and AD patients compared to healthy individuals (25). Most researches have found that regions of the temporal lobe and parietal lobe are often affected in the early stage of MCI, typically manifested as declines in semantic memory and vocabulary retrieval ability. Therefore the semantic fluency task is more sensitive to the early cognitive decline of MCI patients (23, 26). Working memory, regarded as a cornerstone of higher-order cognitive functions, is frequently employed as a paradigm for investigating cognitive processes (27). Certain studies have proposed that prefrontal working memory-related networks could serve as sensitive markers for the early screening of dementia (28). In the n-back task, a series of stimulate are presented and participants decide if the current stimulate is the same as the item presented n positions back, including 0-back, 1-back, 2-back, 3-back. With a higher n value, the heavier the cognitive load (29). Some studies have found that there is a certain correlation between the accuracy and response time of n-back and age, from 0-back to 1-back, there is no increase in age-related differences, but in 2-back or 3-back, an additional cost was accrued in both accuracy and response time (30). The 1-back has a low cognitive load and is suitable for the ability range of patients with MCI, which can effectively detect impairment of working memory in patients with MCI, while avoiding obscuring the true ability due to difficult tasks (31, 32). Furthermore, the analysis of Montreal Cognitive Assessment (MoCA) cognitive subscores from 15 MCI patients in the early stages of our research revealed that the primary cognitive impairments were in visuospatial and executive abilities, language, and delayed memory. Minimal point losses were observed in naming and orientation (Figure 1). These findings suggest that the VFT and n-back paradigms may be more appropriate for screening individuals with early-stage MCI. However, whether the VFT or the 1-back task can earlier identify MCI has not yet been investigated in related studies.

In this study, cerebral activation patterns in the cortical regions were investigated by assessing 103 patients with MCI and 108 healthy individuals using the VFT and 1-back task with fNIRS. The correlation between brain activation channels, regions of interest (ROIs), and cognitive function in MCI patients was also analyzed. Demographic and clinical characteristics of 103 participants in Table 1.

**Table 1:** Demographic and clinical characteristics of participants.

Participants characteristics	MCI		HC		Test statistics
	*N*	Mean (SD)	*N*	Mean (SD)	
Sex (male/female)	40/63		47/61		*χ*^2^(1) = 0.477, *p* = 0.490
Age (years)	103	73.48 ± 5.978	108	72.46 ± 6.579	*t* = 1.169, *p* = 0.244
Education	103	12.88 ± 3.024	108	13.75 ± 3.451	*t* = −1.936, *p* = 0.054
MoCA	103	21.84 ± 2.186	108	26.49 ± 1.580	*t* = −17.755, *P*<0.001
Words (VTF)	103	18.67 ± 5.04	108	21.30 ± 4.50	*t* = −3.876, *P<*0.001
Accuracy (1-back)	103	0.62 ± 0.22	108	0.74 ± 0.21	*t* = −4.033, *P<*0.001
Reaction Time(1-back,ms)	103	811.51 ± 188.73	108	786.66 ± 192.04	*t* = 0.934, *p* = 0.352
SCL-90	103	110.17 ± 20.974	108	112.54 ± 24.885	*t* = 0.747, *p* = 0.456
PSQI	103	7.54 ± 4.313	108	7.54 ± 4.329	*t* = 0.011, *p* = 0.991

## 2 Materials and methods

### 2.1 Ethics statement

The studies that involved human participants were reviewed and received approval from the Human Ethics Committee of the Hospital. Written informed consent to participate in this investigation was procured from all participants.

### 2.2 Participants

A sum of 211 older adults aged 60 years and above were enrolled in this study from community populations. The inclusion criteria included the following: (a) the absence of severe hearing, speech, or comprehension impairments, ensuring participants could successfully complete various cognitive assessments and fNIRS tests; (b) no severe or poorly managed underlying medical conditions, particularly cardiovascular diseases; (c) no documented history of psychiatric illness or the use of psychotropic medications; (d) no record of substance addiction, such as to drugs or alcohol; (e) right-handedness. Informed consent forms were signed by all participants, who were provided with a gift at the conclusion of the experiment. This study was conducted at XX Hospital, with the screening process performed by cognitive rehabilitation physicians at these institutions. The MCI label was given to participants based on NIA-AA and Petersen’s criteria (33, 34). We integrate cognitive function assessments to diagnose MCI Additionally. Cognitive function was primarily evaluated using the MoCA scale (35).

Due to the influence of education level on MoCA scores, the cutoff values for MoCA have been adjusted according to the characteristics of the Chinese population and years of education to ensure the accuracy of the assessment and to avoid misclassifying individuals with high education levels and normal cognitive function as having MCI (36, 37). In this investigation, participants were categorized into the mild cognitive impairment group (experimental group, EG) or the cognitively healthy group (control group, CG) according to the following standards: Individuals were allocated to the MCI group if their education duration was fewer than 7 years with a MoCA score of 21 or below, if their education spanned 7–12 years and their MoCA score was 23 or below, or if their education exceeded 12 years and their MoCA score was below 26. Conversely, in the absence of a clinical diagnosis of MCI or any cognitive impairment, those with fewer than 7 years of education and a MoCA score above 21, those with 7–12 years of education and a MoCA score above 23, or those with more than 12 years of education and a MoCA score of 26 or above were assigned to the cognitively healthy group.

The experimental procedures adhered to the most recent guidelines outlined in the Declaration of Helsinki. Ethical approval for the research was procured from the local Institutional Review Board, and the study was registered in the Chinese Clinical Trials Registry (no. ChiCTR2300071569). To ensure the study had adequate statistical power, *a priori* power analysis was conducted using G*Power software. With a significance level (*α*) of 0.025, statistical power of 0.95, and an expected medium-to-large effect size (Cohen’s *d* = 0.8), the analysis indicated that a total of 98 participants (49 per group) would be sufficient. The actual power achieved with this sample size was 0.952, confirming the adequacy of the sample for detecting the expected effect (38).

Functional near-infrared spectroscopy data were processed using the Homer2 software suite (39), following the analytical framework and parameters outlined by Channels exhibiting excessive light intensity saturation were excluded from the dataset (40). To identify these suboptimal channels, we applied the relative coefficient of variation (CV, expressed as a percentage) as a metric (41). Participants with channels showing a CV channel exceeding 15% were excluded from further analysis. For those with CV channel values below 15%, the channels identified as poor quality were treated as missing data during statistical analyses.

### 2.3 Devices

**Figure 2:**
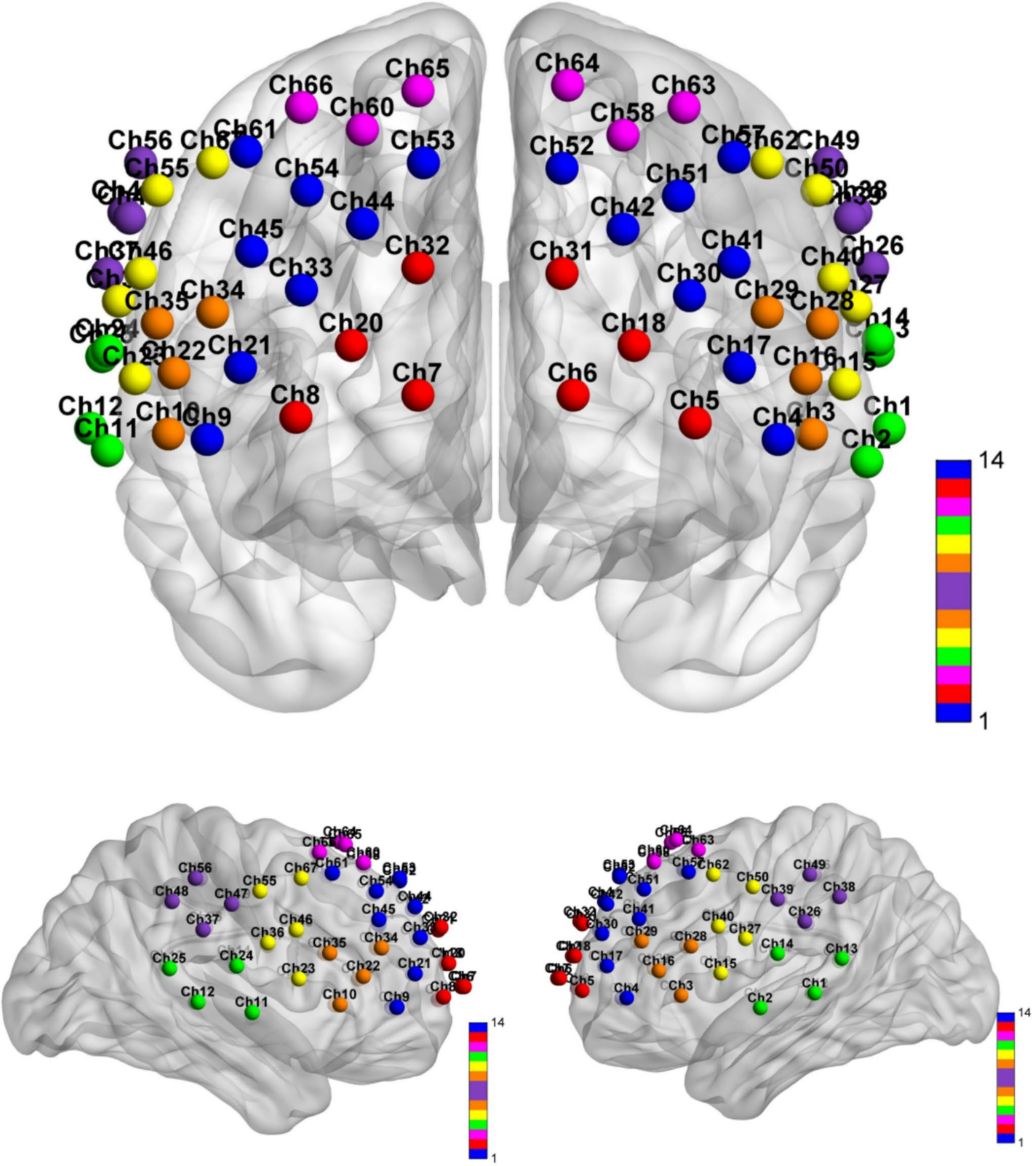
Distribution of channels.

The fNIRS data acquisition was conducted utilizing a BS-7000 system (Wuhan Znion Technology Co., Ltd., Wuhan, China), equipped with dual-wavelength laser diodes (690 and 830 nm) and operating at a 100 Hz sampling rate. This apparatus comprised 16 sources and 16 detectors, with each channel established by a source–detector pair positioned at an inter-optode distance of 3 cm, resulting in 67 channels in total. In accordance with the international 10–20 EEG placement system, the optode S2 was aligned with the Fpz point. Post-transformation of channel coordinates using NIRS-SPM25 facilitated their mapping onto the cortical surface, where they were categorized into 7 ROI based on Brodmann’s areas. This arrangement ensured the symmetrical distribution of channels across both hemispheres, as illustrated in Table 2 and Figure 2.

**Table 2:** Correspondence between 67 channels and Brodmann area.

Area number	Brodmann area	Channel number
1	DLPFC	[4, 17, 30, 42, 51, 52, 57, 43, 61, 53, 54, 44, 33, 21, 9, 41, 45]
2	Temporal	[1, 2, 11, 12, 13, 14, 24, 25]
3	PreM and SMC	[15, 40, 50, 62, 67, 55, 46, 23, 27, 36]
4	Broca	[28, 35, 3, 16, 29, 34, 22, 10]
5	SSC	[39, 26, 49, 56, 37, 47, 38, 48]
6	FPA	[5, 6, 18, 31, 19, 32, 20, 7, 8]
7	FEF	[58, 63, 64, 59, 65, 66, 60]

DLPFC, dorsolateral prefrontal cortex; TC, temporal area; PreM and SMC, premotor and supplementary motor cortex; DHPC, dorsoventral hippocampus; SSC, somatosensory cortex; FPA, frontal pole area; FEF, frontal eye fields.

The evaluation relied on variations in the concentration of oxygenated hemoglobin (HbO) within the relevant cortical areas during the VFT and 1-back tasks. In accordance with the Beer–Lambert law, the optical signals detected by the photodetector were transformed into HbO concentration signals, and the mean value was determined following the completion of the tasks repeated three times.

### 2.4 Experimental procedure

**Figure 3:**
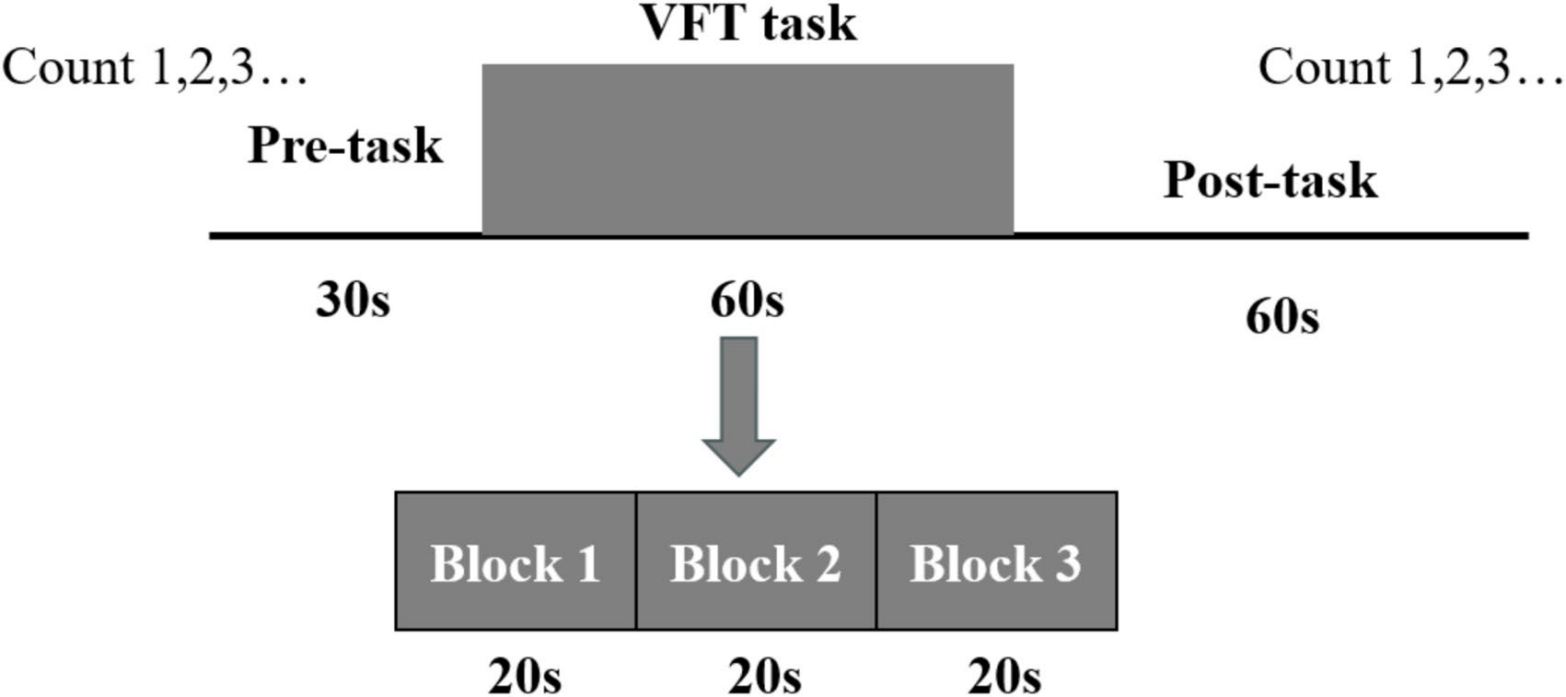
The verbal fluency task design.

This investigation incorporated two tasks, namely the VFT and 1-back task, carried out at the XX Hospital. Prior to initiating the experiment, the staff provided a detailed explanation of the experimental procedures to the participants, beginning with the MoCA evaluation. Based on the resulting scores, participants were categorized into the experimental and control groups. Subsequently, the fNIRS data were acquired. The VFT task was introduced by the staff, who also fitted the participants with a fiber optic cap and performed signal calibration. Initially, a 10-s pre-scanning phase was conducted, during which participants were instructed to count along with a voice broadcast. Following the collection of a 20-s baseline, participants engaged in a 60-s word-generation task. This task comprised three items, with each involving a new word category (e.g., fruits, furniture, colors, and vegetables) every 20 s. A subsequent 60-s relaxation phase followed, consisting of 50 s of voice-guided counting to serve as the baseline phase for neurological and blood flow activity recovery and a concluding 10-s end phase. The entire procedure spanned 150 s, as illustrated in Figure 3.

**Figure 4:**
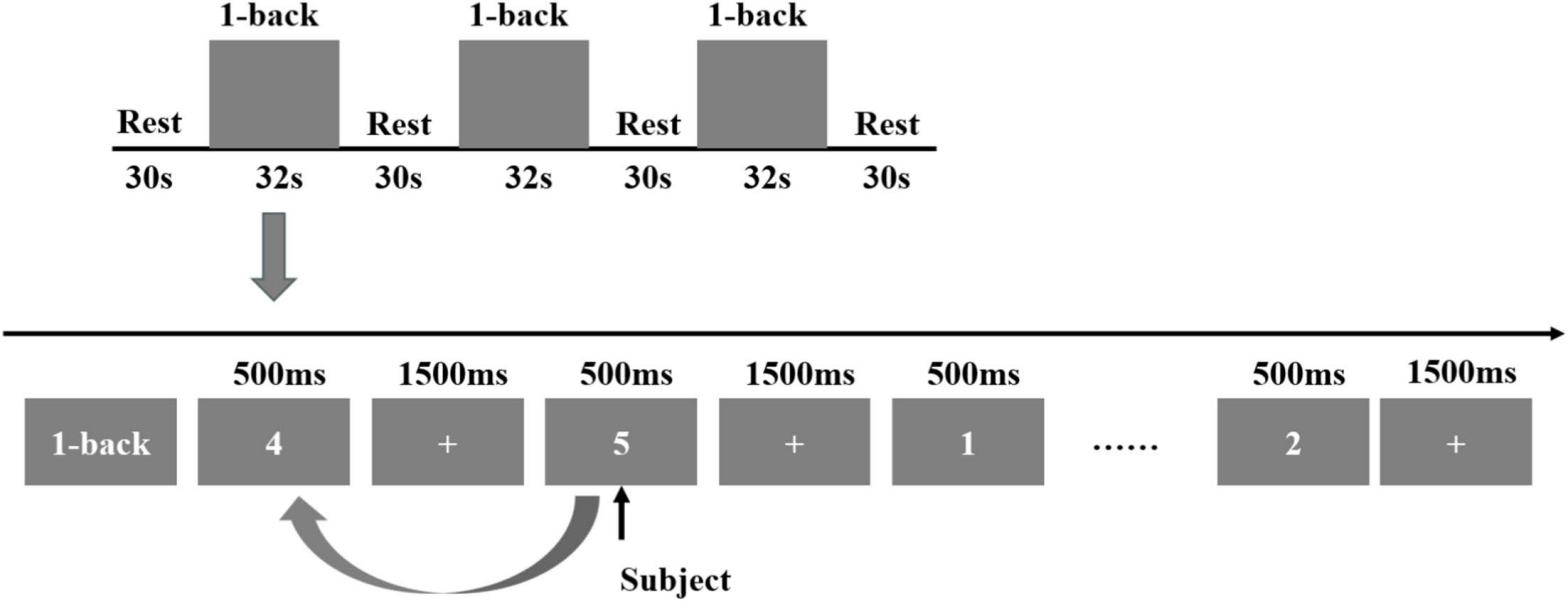
The 1-back task design.

Subsequently, the 1-back task was administered. During this task, a 2-s prompt was initially displayed, followed by a number shown for 500 ms. After each number presentation, a fixation cross appeared for 1,500 ms. The number was presented 15 times, and participants were required to determine whether each stimulus matched the number displayed immediately prior. The task was repeated three consecutive times, after which a 30-s relaxation phase was conducted. Upon completion of the fNIRS data collection, the Symptom Checklist-90 (SCL-90) was employed to evaluate patients’ mental and psychological states, while the Pittsburgh Sleep Quality Index (PSQI) was utilized to assess sleep quality, as illustrated in Figure 4.

### 2.5 Data preprocessing

Data analyses were performed using MATLAB R2014b, integrating the Homer2 toolbox for preprocessing, which comprised the following steps: (1) transformation of raw light intensity into optical density; (2) identification and correction of motion artifacts through a sliding time window (Sdthresh = 20, AMPthresh = 5, tMotion = 0.5 s, tMask = 3 s); (3) implementation of bandpass filters for functional connectivity analysis (0.01–0.1 Hz) to mitigate artifacts such as baseline drift and heartbeat interference (42); (4) computation of changes in HbO, deoxygenated, and total hemoglobin concentrations based on the modified Beer–Lambert law (43); (5) to enhance the reliability of the results, employed block averaging to reduce noise and improve signal detection. For the 1-back task, we averaged the data from each block using a time window from −10 to 50 s relative to the block onset. In contrast, the VFT includes only a single block. For this task, we used a longer averaging interval from −10 to 115 s to capture the full extent of the neural response.

An individual-level analysis was performed using the general linear model (GLM) to identify task-related neural activity. The GLM estimates were derived by calculating the weight coefficients (*β* value) for each task variable, incorporating user-defined contrast vectors to focus on specific conditions of interest (44). This process allowed us to determine the average amplitude of the neural response for group-level analysis. For visualization, we used the BrainNet toolbox to create three-dimensional representations on a standard brain surface. The integrated statistical values and Montreal Neurological Institute (MNI) coordinates for each channel were computed, with the brain surface colors reflecting the t-statistic values from the group-level *t*-test.

### 2.6 Statistical analysis

Statistical analysis of the data in this study was executed utilizing SPSS 25.0 software. Initially, all data were examined for normality. Differences in sex were evaluated via the chi-square test, while independent-sample *t*-tests were applied to compare other demographic and cognitive variables, including MoCA scores, age, and education level. A significance threshold (*α*) of 0.05 was established for all statistical tests. Results were deemed statistically significant if the *p*-value was ≤ 0.05 and highly significant if the *p*-value was ≤ 0.01. Count data were summarized using frequencies, percentages, or component ratios. Measurement data were denoted as mean ± standard deviation or as the median. For data adhering to a normal distribution, one-way ANOVA was employed, whereas the rank sum test was utilized for non-normally distributed data, and the chi-square test was applied for count data. Pearson’s correlation coefficients were calculated to evaluate the link between the clinical screening tool and hemodynamic signals.

## 3 Results

### 3.1 Comparison of channel hemodynamic responses

**Figure 5:**
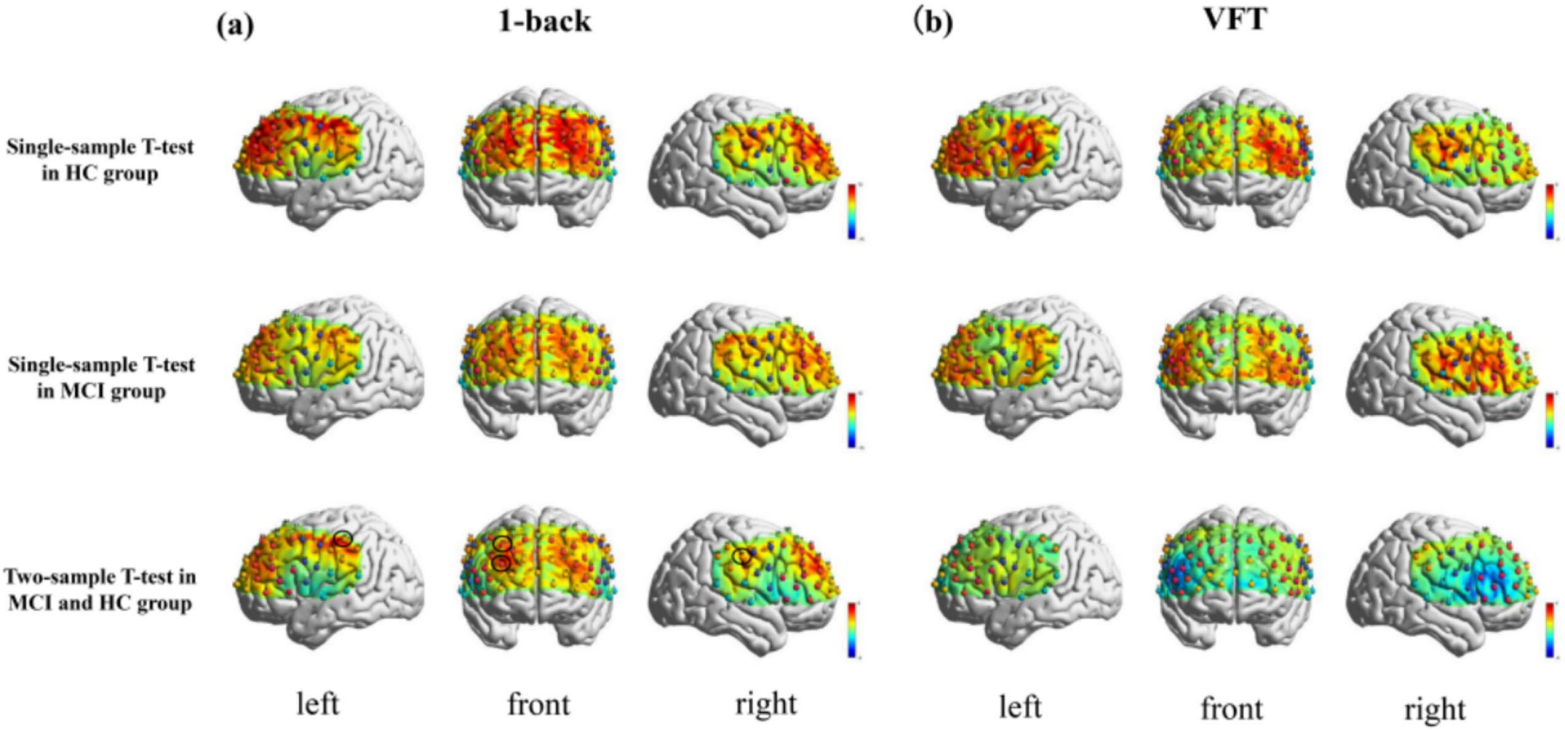
Activation maps of HbO signal increase during 1-back task and VFT. Among them, Ch33, Ch47, Ch49, Ch54 was found activated during the 1-back task and no channels were found activated in any of the VFT task. **(a)** 1-back task. **(b)** VFT.

The t-map and correlation map are extensively utilized as image markers in the domain of fNIRS. Figure 5 presents the group-averaged t-maps derived from 67 channels for MCI patients and HC during two tasks, namely the 1-back task and the VFT. This analysis aims to examine the activated channels in MCI patients compared to HC. The figures illustrate the average HbO levels for each group.

The results indicated a notable increase in HbO signals in four channels during the 1-back task, whereas no significant increase was detected in any channels during the VFT.

### 3.2 Comparison of ROI hemodynamic responses

**Figure 6:**
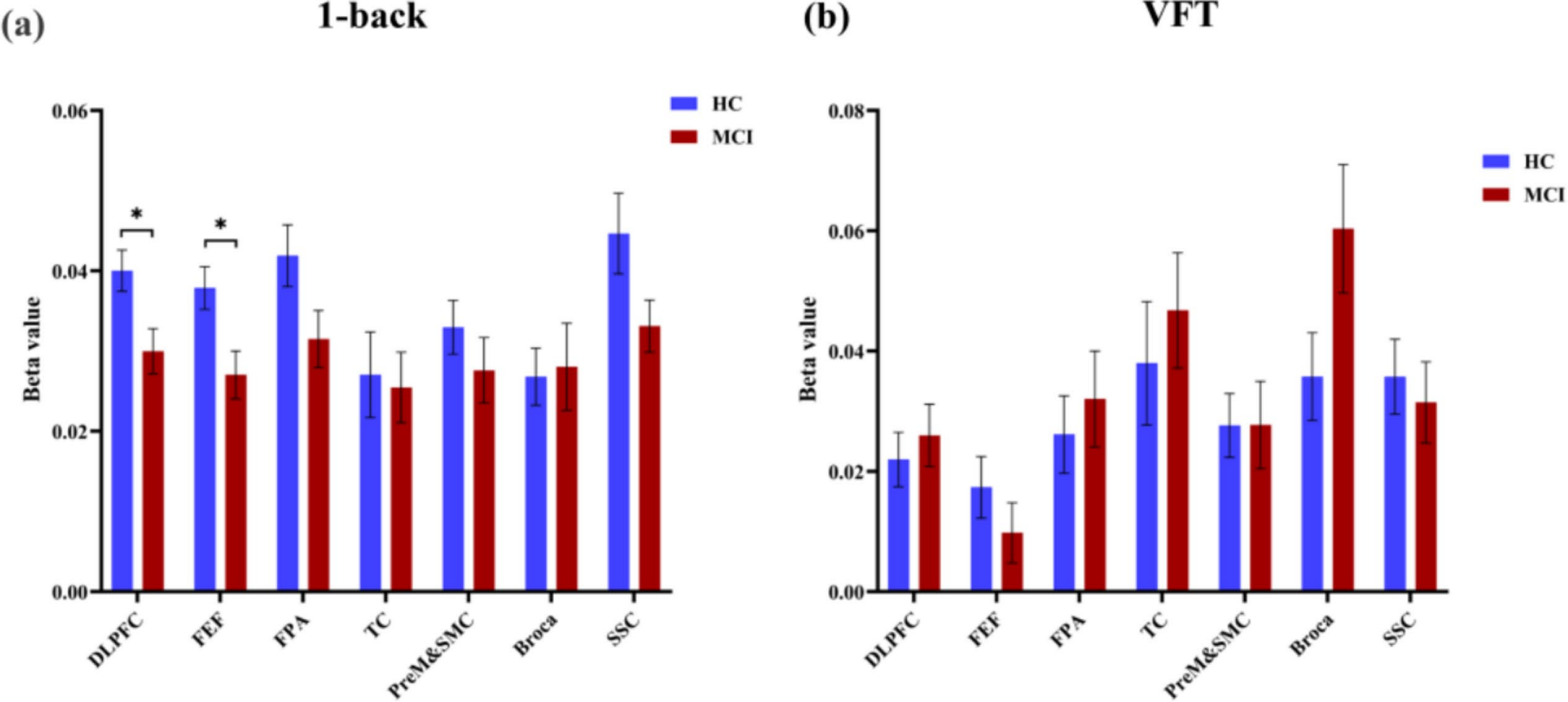
Activated ROIs for 1-back task. **(a)** 1-back task, *during the 1-back task, the MCI group exhibited significantly reduced activation levels in DLPFC and FEF(*p* < 0.05). **(b)** VFT.

**Figure 7:**
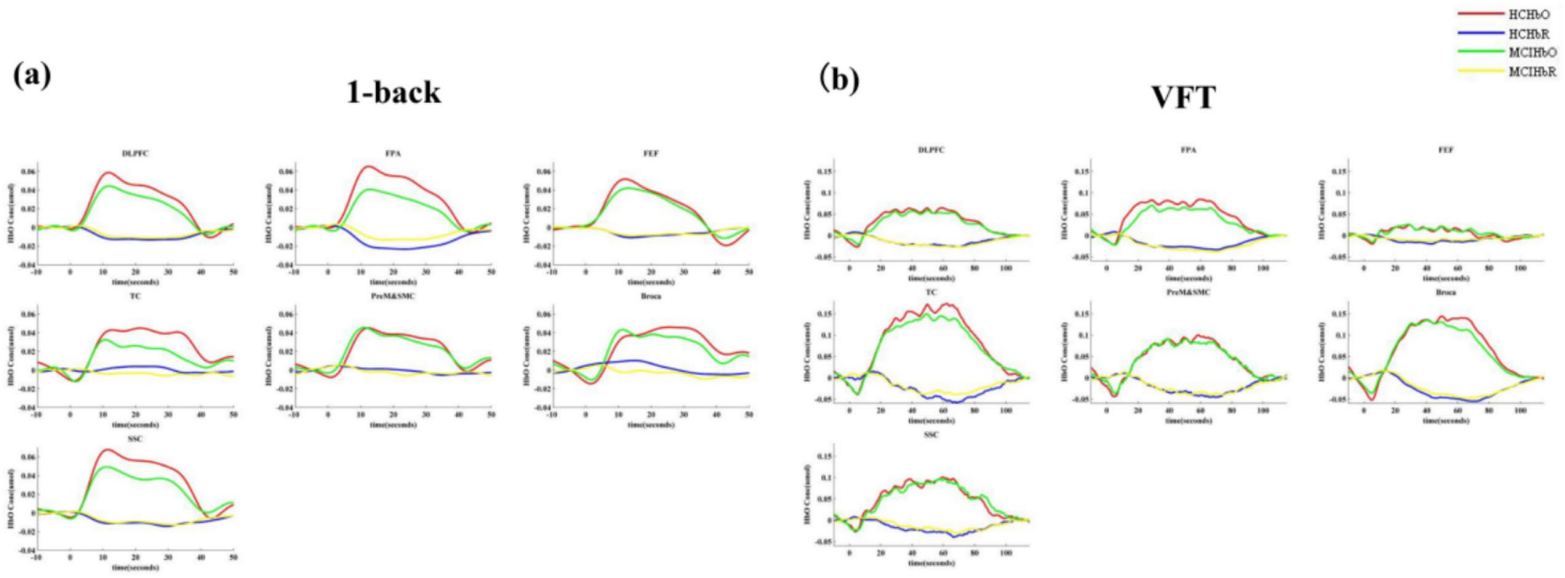
The overall oxy- and deoxy-Hb signals in ROIs. **(a)** 1-back task. **(b)** VFT.

The findings revealed a notable increase in HbO signals within the DLPFC and FEF during the 1-back task, whereas no significant activation was detected in any ROI during the VFT. Figure 6 illustrates the locations of ROIs exhibiting significant activation during the 1-back task. Figure 7 depicts the averaged HbO signals recorded in these activated channels across participant groups, providing an overall representation of the signals for the ROIs during the 1-back task and VFT. Table 3 presents the statistically significant between-group differences in GLM *β* values across Ch33, Ch47, Ch49, Ch54, as well as in the DLPFC and FEF during the 1-back task.

**Table 3:** Significant results of between-group differences in GLM-β values for the 1-back task.

Channel/ROI	HC (M ± SD)	MCI (M ± SD)	*p*-value	FDR *P-*value	*T*-value	HC vs. MCI
Ch-33	0.05 ± 0.04	0.03 ± 0.03	*p* = 0.002	*p* = 0.026	3.30	HC > MCI
Ch-47	0.05 ± 0.04	0.03 ± 0.04	*p* = 0.003	*p* = 0.045	3.04	HC > MCI
Ch-49	0.06 ± 0.04	0.03 ± 0.04	*P* < 0.001	*p* = 0.023	3.59	HC > MCI
Ch-54	0.05 ± 0.04	0.03 ± 0.04	*P* < 0.001	*P* = 0.023	3.45	HC > MCI
DLPFC	0.04 ± 0.04	0.03 ± 0.03	*p* = 0.010	*p* = 0.032	2.63	HC > MCI
FEF	0.04 ± 0.03	0.03 ± 0.03	*p* = 0.008	*P* = 0.032	2.72	HC > MCI

### 3.3 Correlation analysis of channels, ROIs and MoCA

**Figure 8:**
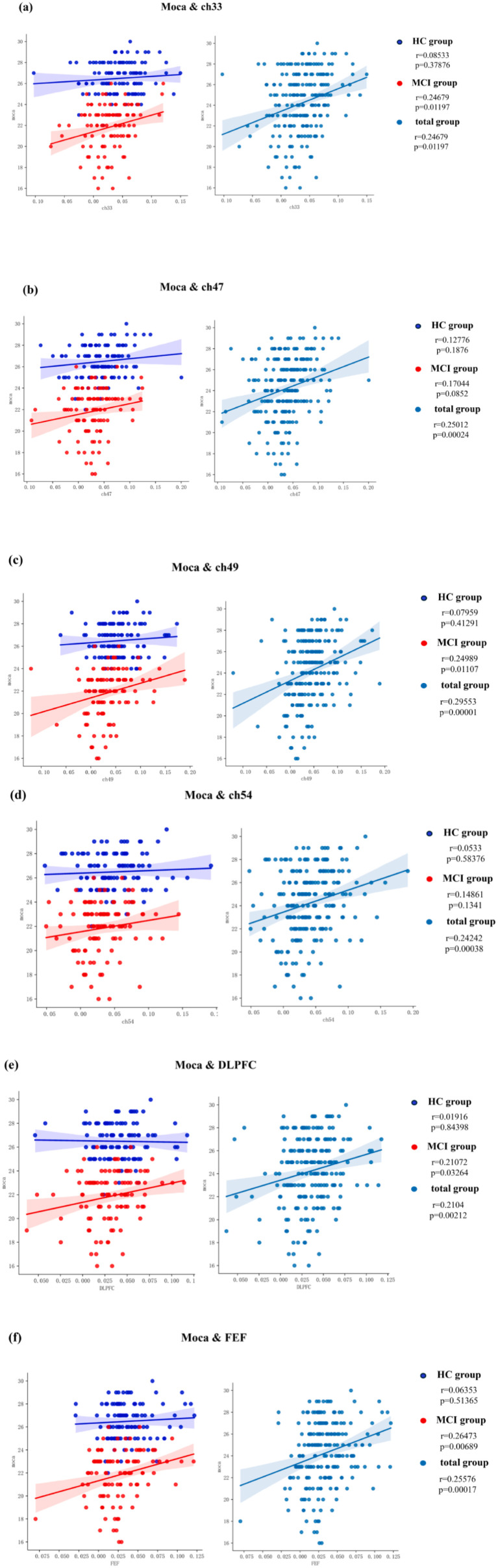
Correlation analysis of channels, ROIs and MoCA. **(a)** MoCA and Ch33, **(b)** MoCA and Ch47, **(c)** MoCA and Ch49, **(d)** MoCA and Ch54, **(e)** MoCA and DLPFC, **(f)** MoCA and FEF.

Simple linear regression was utilized to examine potential correlations between fNIRS parameters (beta value), specifically Ch33, Ch47, Ch49, Ch54, DLPFC, and FEF, and behavioral data (MoCA scores). Notably, a moderate positive correlation (*R* ≥ 0.5) was identified between one of the fNIRS parameters and the MoCA score in MCI patients, whereas no such correlation was observed in HC, as depicted in Figure 8.

## 4 Discussion

This study sought to investigate alterations in brain activation patterns during the VFT and 1-back task in individuals with MCI and healthy controls, along with the relationships between brain activation channels, ROI, and cognitive function in MCI patients. Furthermore, the research seeks to determine a more appropriate fNIRS experimental paradigm for individuals with MCI. Hemodynamic properties were examined in both healthy participants and those with MCI. fNIRS signals were recorded from the brain during the VFT and 1-back task to detect differences in activated regions between healthy controls and individuals with MCI. The findings revealed that fNIRS-related indicators, specifically HbO levels in Ch-33 and Ch-49, as well as in the dorsolateral prefrontal cortex (DLPFC) and frontal eye fields (FEF), during the 1-back task exhibited a significant positive correlation with MoCA scores.

Mild cognitive impairment-related atrophy has been associated with changes in both the anatomical structures and functional organization of the dorsolateral prefrontal cortex (45) and frontal eye fields, which influence the metabolic activity of cortical neurons in these regions (46, 47). Such alterations can be identified using brain imaging techniques (10, 48). Recent fNIRS studies have aimed to detect specific brain signals linked to MCI, revealing hemodynamic changes in the cerebral cortex of affected individuals. Prior research has consistently demonstrated markedly reduced activation in the left frontal, right superior frontal, and left temporal lobes in MCI patients during cognitive tasks (45, 49). Similarly, in the present study, lower activation was observed in the bilateral prefrontal, parietal, and occipital cortices in MCI patients compared to healthy controls (10, 23, 50). These findings indicate that variations in blood flow response patterns between healthy aging and pathological aging can be explored through fNIRS, aligning with earlier research (49, 50). These differences may stem from neurodegenerative processes that inhibit neural activities in the left dorsolateral prefrontal cortex, left anterior motor area, supplementary motor area, and frontal eye fields, resulting in insufficient neural resource recruitment for cognitive tasks in individuals with MCI (49). Specifically, the decline of gray matter in the cerebral cortex, which is evident in MCI patients, may contribute to a relative reduction in HbO levels in the cerebral cortex during VFT and working memory tasks compared to healthy controls (51).

The n-back task is widely regarded as one of the most frequently employed working memory (WM) paradigms in cognitive neuroscience. The 1-back task encompasses a variety of cognitive processes, including lower-order perceptual and motor functions such as visuospatial attention and response selection, alongside higher-order control mechanisms like resistance to proactive interference and the updating and monitoring of working memory. Verbal fluency tasks are extensively utilized in both clinical and research settings to assess lexical access speed and executive functions, particularly those related to updating, inhibition, and mental flexibility (52). These findings highlight the well-established link between verbal fluency and MCI (53, 54). In earlier stages of research, numerous studies applied the n-back and VFT tasks for MCI screening (25, 29). In this study, it was notably observed that mHbO levels in the dorsolateral prefrontal cortex and frontal eye fields were markedly reduced during the 1-back task. While a downward trend in HbO was apparent in certain ROIs during the VFT, no statistically significant differences were detected between HC and MCI patients. During the 1-back task, the MCI group exhibited statistically significant changes in GLM metrics across multiple channels, suggesting that this task may possess higher sensitivity in fNIRS studies for reflecting alterations in cognitive status. Compared to the VFT task, the 1-back task demonstrated more pronounced between-group differences, indicating its potential suitability as a paradigm for fNIRS research to investigate neural activity patterns associated with MCI. This discrepancy may arise from the fact that cognitive decline in MCI patients is not solely reflected in speech and executive function but is likely indicative of multidimensional impairments in memory, attention, reflexes, calculations, and visuoconstruction (55). The DLPFC, a core brain region for working memory (56), and the FEF which are critically involved in visual attention and task switching (57), collectively suggest that individuals with MCI demonstrate early-stage declines in both working memory performance and attentional control capabilities. The VFT predominantly engages Broca’s area (58) and the semantic memory network (59), regions that are typically less affected in the early stages of MCI. Consequently, the 1-back task, which places greater demands on working memory and attentional control, demonstrates higher sensitivity in detecting early cognitive impairments in MCI patients. Compared to other neuroimaging modalities such as fMRI and PET, fNIRS demonstrates distinct advantages, including lower procurement and maintenance costs, reduced detection times, and the capacity for rapid data acquisition and analysis. These features render fNIRS particularly advantageous for the early screening of MCI in primary care and community hospital settings. By employing the 1-back task, fNIRS can effectively identify alterations in brain activity associated with working memory and attentional control in MCI patients, enabling the early detection of high-risk individuals. This approach is highly suitable for large-scale implementation, offering a cost-effective and efficient solution for MCI screening.

Interestingly, we observed significantly diminished activation levels in Ch-47 and Ch-49 during 1-back task performance in MCI patients. Both channels are localized within the somatosensory cortex (SSC) based on standardized brain atlas registration. However, ROI analysis revealed no statistically significant reduction in overall SSC activation. Early studies conventionally suggested that primary sensory functions might be preserved in AD (60). However, emerging evidence indicates that somatosensory dysfunction is prevalent among AD patients, with these deficits often masked by more prominent cognitive impairments (61). Our findings suggest that somatosensory dysfunction may represent an early marker in MCI. The observed pattern implies that the 1-back task may lack sufficient sensitivity for detecting early SSC alterations. For future research, particular emphasis should be placed on investigating the impact of SSC on cognitive functions, while concurrently developing more sensitive assessment tools for evaluating somatosensory dysfunction.

Prior research has demonstrated that sleep disorders and mental states can influence cognitive function (62, 63). Consequently, this study also evaluated sleep quality and emotional state in MCI patients and healthy older adults, revealing no significant differences between the two groups. It was observed that more severe insomnia symptoms were linked to poorer performance in global cognition as well as immediate and delayed logical memory recall, particularly when combined with short sleep duration (64). This phenomenon may stem from significant alterations in synaptic structure and signaling pathways associated with sleep disorders, including the weakening of AMPA receptor function through dephosphorylation. Homer 1a, a crucial factor in sleep–wake regulation, contributes to reduced excitatory synaptic function during sleep, thereby facilitating synaptic remodeling and memory consolidation (65). Mental status is similarly closely associated with cognitive impairment. Neuropsychiatric symptoms have been identified as strongly correlated with cognitive deficits, especially during the early stages of AD. Common symptoms include depression, anxiety, and apathy, while delusions and irritability are considered independent risk factors for cognitive decline. Some studies have indicated that neuropsychiatric symptoms are predominantly linked to impairments in executive control and reductions in gray matter volume in the orbitofrontal and posterior cingulate cortices (66). Although significant differences were not identified between the two groups, sleep disturbances and psychological sub-health were evident in both. Thus, when employing fNIRS for the early diagnosis of MCI, sleep quality and mental state should remain areas of focus.

In this study, fNIRS was employed to capture cortical hemodynamic responses in MCI patients during the VFT and the 1-back task. The findings indicate that the 1-back task may demonstrate superior sensitivity in detecting early cognitive alterations in MCI within the context of fNIRS-based research. Nevertheless, this study is subject to several limitations. First, the analysis was predominantly confined to task-evoked data and cortical activation profiles, without investigating alterations in functional connectivity networks. Future investigations should delve into network-level connectivity changes to achieve a more holistic understanding. Second, although the combined analysis of oxygenated HbO and deoxygenated hemoglobin (HbR) can provide a more comprehensive depiction of neurovascular coupling, this study did not incorporate HbR data. Subsequent research should encompass a dual analysis of both HbO and HbR to enhance the robustness of the findings. Lastly, we intend to undertake longitudinal follow-up studies to further assess the clinical applicability of the 1-back task in the early detection and progression monitoring of MCI.

## 5 Conclusion

This study sought to assess fNIRS-derived signals as a potential biomarker for MCI. To this end, hemodynamic properties were examined in both healthy participants and individuals diagnosed with MCI. fNIRS signals were recorded from the brain during the VFT and 1-back task to identify differences in activated regions between healthy controls and individuals with MCI. Notably, during 1-back tasks, MCI patients exhibited reduced brain activity levels in the dorsolateral prefrontal cortex and frontal eye fields. This task-induced decrease in brain activity was found to be markedly positively correlated with MoCA scores and demonstrated minimal influence from mental state and sleep quality. Compared to the VFT, the 1-back task appears to be a more effective paradigm for the early detection of MCI.

## Data Availability

The raw data supporting the conclusions of this article will be made available by the authors, without undue reservation.
