# Supplementary material for: An fNIRS-based investigation of cerebral hemodynamic responses during verbal fluency task and n-back task in individuals with mild cognitive impairment
Source: Front Neurol. 2025 May 8;16:1571964. doi: 10.3389/fneur.2025.1571964 (PMC12094942; doi:10.3389/fneur.2025.1571964)
Supplement: Supplementary file 1 [file Table_1.docx]

We analyzed the HbR index of the MCI group and the HC group under the 1-back and VFT paradigms, and found that only the HbR of the CH-54(*P*＜0.05) and DLPFC(*P*＜0.05) of the 1-back was significantly decreased, as illustrated in Table 1.

Table 1. Significant results of between-group diferences in HbR for the 1-back task

| Channel/ROI | HC(M±SD) | MCI(M±SD) | *P* value | FDR *P* value | T value | HC vs MCI |
| --- | --- | --- | --- | --- | --- | --- |
| Ch-54 | 0.016±0.022 | 0.007±0.021 | *P* < 0.001 | *P*=0.003 | 4.14 | HC＞MCI |
| DLPFC | 0.014±0.012 | 0.008±0.014 | *P*=0.002 | *P*=0.009 | 3.25 | HC＞MCI |
