# Supplementary material for: An fNIRS-based investigation of cerebral hemodynamic responses during verbal fluency task and n-back task in individuals with mild cognitive impairment
Source: Front Neurol. 2025 May 8;16:1571964. doi: 10.3389/fneur.2025.1571964 (PMC12094942; doi:10.3389/fneur.2025.1571964)

Logistic Regression model was used to classify subjects based on their group membership. The model was evaluated using Leave-One-Out Cross Validation (LOOCV). The logistic regression model was regularized with L2 penalty and C = 0.01, and the 'liblinear' solver was used for optimization.

The Logistic Regression model was evaluated on two cognitive tasks: the 1-back task and the VFT task. A illustrates the accuracy of the model on the two tasks, showing that the 1-back task achieved a higher accuracy of 0.64, while the VFT task had a lower accuracy of 0.57. Plot B shows the AUC values, where the 1-back task again outperformed the VFT task, with an AUC of 0.66 compared to 0.51 for the VFT task.


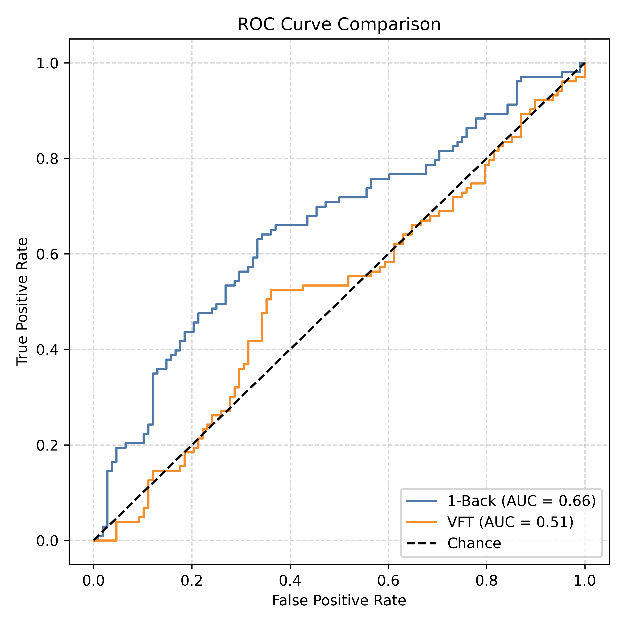

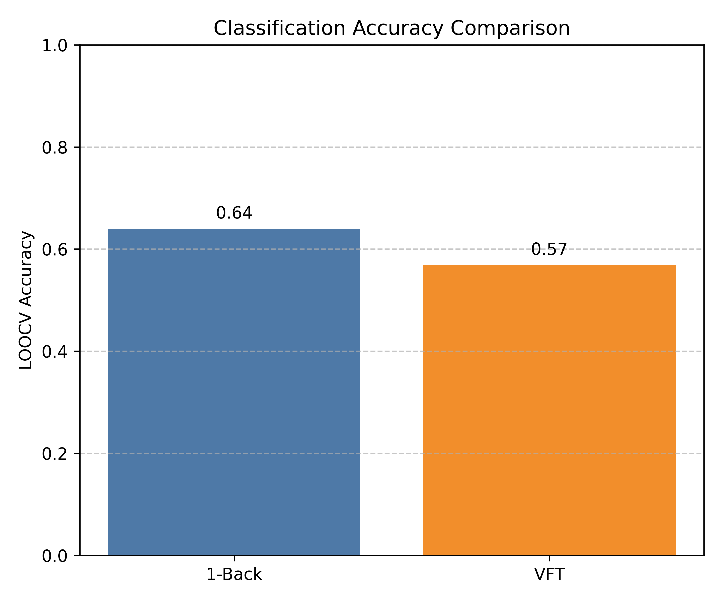

Supplement: Supplementary file 2 [file Table_2.docx]
